# Study of a novel front-roof-back natural ventilation system for Chinese solar greenhouses

**DOI:** 10.1098/rsos.220251

**Published:** 2022-11-16

**Authors:** Lei Zhang, Xingan Liu, Wenbin Shi, Tianlai Li, Jianwei Ji

**Affiliations:** ^1^ College of Information and Electrical Engineering, Shenyang Agricultural University, No. 120 Dongling Road, Shenhe District, Shenyang 110866, People's Republic of China; ^2^ College of Horticulture, Shenyang Agricultural University, No. 120 Dongling Road, Shenhe District, Shenyang 110866, People's Republic of China; ^3^ National and Local Joint Engineering Research Center of Northern Horticultural Facilities Design and Application Technology (Liaoning), No. 120 Dongling Road, Shenhe District, Shenyang 110866, People's Republic of China; ^4^ Key Laboratory of Protected Horticulture, Shenyang Agricultural University, Ministry of Education, No. 120 Dongling Road, Shenhe District, Shenyang 110866, People's Republic of China

**Keywords:** solar greenhouse, ventilation, numerical simulation, temperature field, airflow field

## Abstract

A Chinese solar greenhouse (CSG) is a highly efficient and energy-saving horticultural facility. Ventilation is significantly important for crop production in the greenhouse, and the vent configuration is the basis of the greenhouse design. Current CSG ventilation structures mostly include front bottom vents and top vents to create a suitable temperature environment for the normal development of crops. However, the ventilation capacity and efficiency are limited. In the present study, we proposed a comprehensive front bottom + top + back roof (FTB) ventilation configuration. The greenhouse ventilation was investigated during the summer season by means of field testing and simulation, and the performance of three ventilation structures—front bottom + top (FT), front bottom + back roof (FB) and FTB—was compared. The results showed that FTB stabilized the greenhouse temperature for 20 s less time than FT and FB. The cooling rate of FTB showed a 24.84% and 5.52% improvement over FT and FB, respectively, and the average temperature showed a 13.81% and 3.65% decrease, respectively. Moreover, the ventilation performance of the side walls was investigated in order to determine if they might serve as auxiliary structures for FTB ventilation. Nevertheless, the improvements of cooling rate, wind speed and average temperature were only 0.52%, 2.09% and 0.11%, respectively. The results demonstrated that the novel FTB ventilation proposed in the present study significantly improved ventilation efficiency and uniformity compared with conventional ventilation structures. The results presented herein provide theoretical support for the use and design of greenhouses suitable for China's special climate.

## 1. Introduction

A Chinese solar greenhouse (CSG) is characterized as having low cost, excellent thermal insulation performance and high energy efficiency, compared with other greenhouses [1]. The outdoors temperature reaches about −30°C, but these facilities can still ensure a suitable temperature for plants relying on sunlight only during the winter in northeast China [2]. Facility horticulture data in China have shown that the total area of solar greenhouses in China was 4.76 million hectares in 2020, of which about 3.7 million hectares were used for vegetable crops, representing 78% of the total area. Effective control of the solar greenhouse environment is crucial for the healthy development of vegetable crops, as well as for the entire eco-agricultural system [3]. Ventilation is an important strategy for indoor and outdoor energy exchange in a CSG, and it has a direct impact on the temperature and humidity of the greenhouse. It is important to ensure a suitable temperature for plants. The main object of our study is to provide a novel ventilation structure, which effectively improves the cooling rate and increases the ventilation of the plant canopy. In this way, a certain suitable temperature is obtained to maintain the maturity of greenhouse plants. The non-uniform microclimate in greenhouses has attracted the attention of many experts. Overheating and high humidity are harmful to crop production inside the greenhouse, since these conditions may damage plant growth and development [4]. Therefore, in production practices, air exchange plays a pivotal role in maintaining the proper environment inside the greenhouse. Ventilation can reduce the temperature difference between inside and outside air, and decrease humidity and eliminate harmful gases inside the greenhouse [5].

Currently, two types of ventilation pattern are used in the greenhouse, namely natural ventilation and mechanical ventilation. The air circulation between outside and inside makes the temperature in the greenhouse more uniformly distributed, eliminates extreme temperatures, adjusts the microenvironment and helps prevent diseases. Air exchange also promotes air circulation in the greenhouse [6]. In recent years, several researchers have conducted studies on internal environment regulation through mechanical ventilation. They studied various cooling measures including wet curtain and mist cooling, which have been confirmed to have a great impact on the cooling of greenhouses in summer [7]. Fidaros *et al*. [8] studied the effect of mechanical ventilation on greenhouses when they were subjected to solar radiation. In this case, researchers considered the drag force and buoyancy effects of plants. Flores-Velazquez *et al*. [9] analysed the air exchange and temperature distribution in the greenhouse when mechanical ventilation was combined with natural ventilation. Moreover, these researchers designed a more efficient mechanical ventilation system. However, mechanical ventilation consumes a lot of energy and requires a high initial investment, which is not consistent with the low cost of characteristically energy-saving CSGs. Therefore, it is necessary to investigate different aspects of natural ventilation in order to obtain additional knowledge that will help in achieving proper cooling for greenhouses in China.

Natural ventilation plays an important role in CSG technology, and the vent configuration is the key element in natural ventilation design [10]. Natural ventilation is an effective way to maintain a proper microclimate in the greenhouse and also reduces the amount of energy required to operate mechanical ventilation [11]. In addition, outside air is also essential to refurbish the carbon dioxide consumed by plants during photosynthesis [12]. Teitel *et al*. [13] used CFD to study the ventilation efficiency of a mono-span greenhouse. These researchers established relational expressions and concluded that an increased external wind speed led to an increase in the external–internal air exchange rate and decreased the ventilation exchange efficiency of the greenhouse. Bartzanas *et al*. [14] simulated the airflow field and temperature field of different vent structures in a mono-span greenhouse using CFD and suggested that the results using roof + side vents were superior to those when only the roof or side vents were used. In addition, the better the cooling effect, the worse the greenhouse temperature uniformity. Kittas *et al*. [15] used CFD to study the impact of different ventilation configurations including rolling shutter and spin window on greenhouse dehumidification. They concluded that spin window ventilation provided the best greenhouse dehumidification results in terms of energy conversion. Baglivo *et al*. [16] suggest that the optimal choice of glass must be combined with effective scheduling of openings for natural ventilation to avoid internal overheating phenomena. Greenhouse free-cooling control and air flows strongly depend on the volume discretization [17]. These researches required demanding tools to accurately assess the greenhouse cooling and heating energy needs. Most studies on natural ventilation have focused on multi-span greenhouses and dome-shaped greenhouses. CSGs are mostly applied in northern China where the external environment dramatically changes. They have a special structure where heat is stored and released by the walls, which is radically different from multi-span greenhouses and round-arch greenhouses. Therefore, the research results of ventilation cannot be applied here. High-performance natural ventilation must be designed with respect to the structural characteristics of CSG.

In the CSG, natural ventilation is usually the preferred energy-saving strategy. It plays an important role in controlling the internal climate, since it directly affects the internal and external heat exchange and reduces humidity in the greenhouse. The layout of the vents has a significant influence on greenhouse ventilation. Various studies have shown that a good ventilation structure contributes to the exchange of air with the external environment and has an important influence on the cooling, dehumidification and ventilation processes of the greenhouse. A combination of bottom and top natural ventilation may effectively reduce indoor crop diseases. Benni *et al*. [18] conducted field tests and CFD simulations and revealed that closing the windward roof vents resulted in the best natural ventilation for cooling in summer. Akrami *et al*. [19] pointed out that adjusting the vent openings had a great impact on greenhouse crop production. Ganguly & Ghosh [20] demonstrated that the size and location of vents were key factors in designing efficient natural ventilation schemes. The vent configuration directly affected ventilation performance and ventilation efficiency. However, CSG ventilation structures usually include front bottom only, top only, or combined front bottom + top ventilation. Some greenhouses further used mechanical ventilation on the side walls. However, low ventilation performance has been obtained. Thus, these configurations may take advantage of the structural features of the CSG to achieve high-efficiency and high-performance ventilation.

In the present study, we proposed a novel ventilation structure for the CSG. This structure may maximize the cooling effect through the combination of the front bottom, top and back roof natural ventilation. Furthermore, we evaluated the temperature field and velocity field of this ventilation structure by combining experimental testing and numerical simulation under environmental conditions typical of Shenyang in China. An additional advantage of the newly proposed structure is that the FTB ventilation structure can prevent rainwater from entering the greenhouse through the roof vent on cloudy and rainy days, reducing potential damage to the plants.

## 2. Materials and methods

### 2.1. Experiment environment

**Figure 1.**
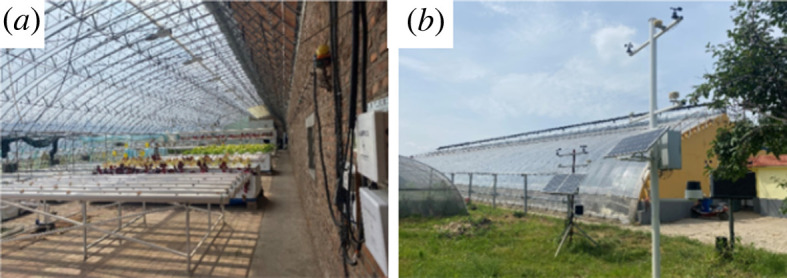
The Liaoshen third-generation greenhouse.

The greenhouse used in this study was a third-generation energy-saving solar greenhouse (figure 1), located at Shenyang Agricultural University (latitude: 41°49′ N, longitude: 123°34′ E, altitude: 42 m). The inside and outside temperatures of the greenhouse were measured on continuously sunny days. The experimental greenhouse was 7° from south to west, with a width of 10 m and a length of 60 m (from east to west), a ridge height of 5.5 m, a north wall height of 4 m, a horizontal projection length of the back roof of 1.9 m, and a wall thickness of 0.5 m for both side and back walls. In addition, a 0.1 m thermal insulation benzene board covered the outside walls, and polystyrene film with a thickness of 0.15 mm covered the front roof of the greenhouse.

**Figure 2.**
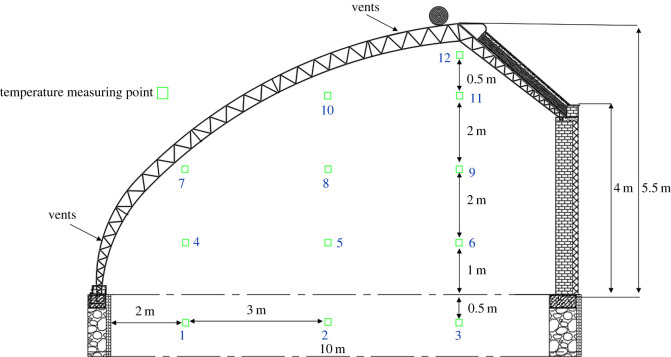
A section of the Liaoshen third-generation greenhouse.

The air temperature and humidity as well as soil temperature were measured. The soil measuring points were located 0.5 m below the soil on the same plane, and 2 m, 5 m and 8 m from the front. Air temperature and humidity were measured at nine points distributed on four different planes. These points were located 2 m, 5 m and 8 m from the front roof on the *x*-axis, with a height of 1 m, 3 m, 5 m and 5.5 m from the ground. The sampling time interval was 10 min. The detailed measuring points are displayed in figure 2. The experimental equipment used in the tests includes RC4HC thermohygraphs, automatic recording devices, data acquisition devices and heat flux sensors. The temperature was measured by the RC4HC thermohygraphs. The instrumental error was lower than ±0.5°C, the resolution was lower than ±0.1°C and the measuring range was −40°C to 80°C. The HOBO, which is made by Onset in America, can measure and store the data. It can also send the data to computers and output the data as an Excel file. The automatic recording device can record the data once per minute, and the temperature error can be controlled within 0.2°C. Equipment probes must be given radioprotection when exposed to air.

### 2.2. Simulation model and calculation method

**Figure 3.**
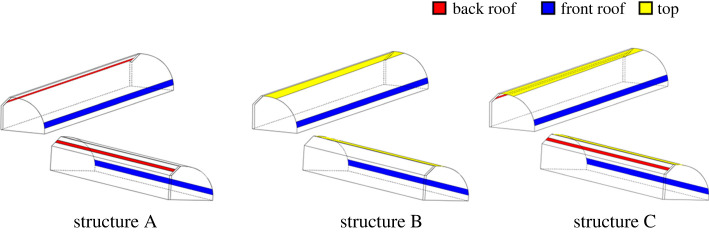
Three-dimensional geometric model of the greenhouse.

The greenhouse ventilation used in this study corresponded to front + top ventilation. SolidWorks was used to build the greenhouse model, with the *x*-axis pointing east, the *y-*axis pointing to the top, the *z*-axis pointing south and the origin located in the northeast corner of the greenhouse. The conventional ventilation of the greenhouse corresponded to front + top ventilation. The top vent was 0.7 m wide. The front vent was 12 m wide and was located 1.5 m from the ground. Nevertheless, in the present study, three different ventilation structures were designed: A (front bottom + top ventilation, FT), B (front bottom + back roof ventilation, FB) and C (front bottom + top + back roof ventilation, FTB). The model is shown in figure 3.

In the present study, the external flow field was considered. The selected dimensions for our model were 100 m × 100 m × 50 m. This model was input into ICEM for trimming, including surface adjustment, as well as part and body creation. In the drawing project, we selected the 1:1 scale. In ANSYS Fluent, the model was resized to match the actual dimensions of the greenhouse. Since this study investigated the natural ventilation of the greenhouse, the energy exchange between the greenhouse and the external environment was an important factor. For the simulation, the setting of the computational domain was an important indicator. Therefore, the geometry model of the greenhouse was built at the horizontal centre of the computational domain.

**Figure 4.**
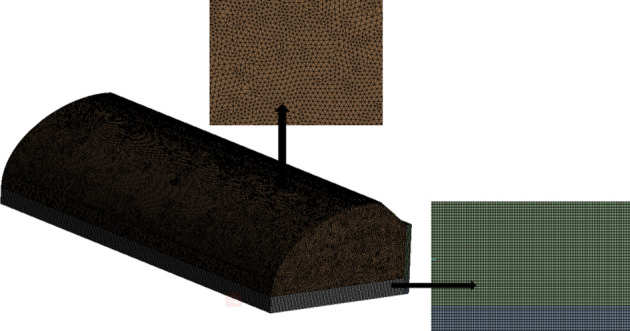
Grid independence analysis.

**Figure 5.**
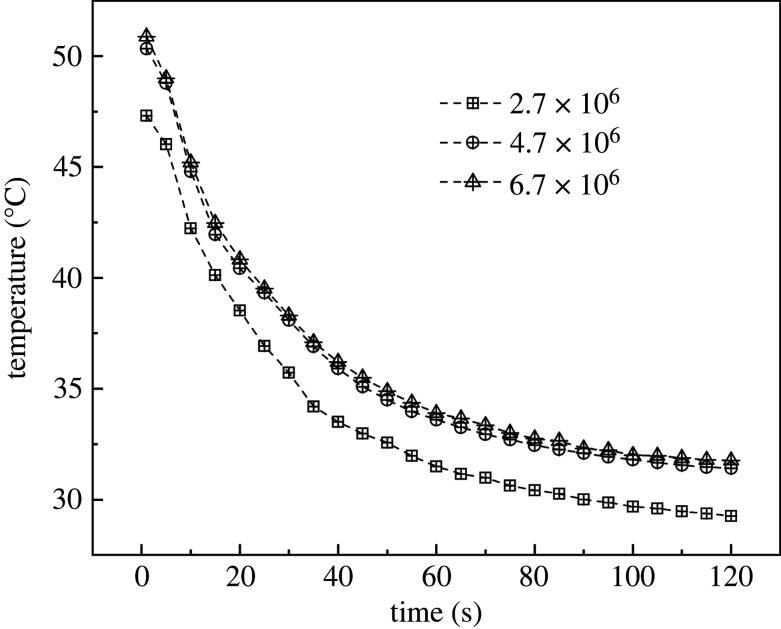
Grid division diagram.

The whole greenhouse and a part of the space outside the greenhouse were set as the calculation range to improve simulation accuracy. The ANSYS Meshing was used to divide the geometry. First, the model was imported and placed at the centre of the computational domain. Second, the model was scaled up to fit the real size of the greenhouse. Third, sizing and meshing were performed to conduct griding with a threshold of 25 mm. The meshing was refined for top and side vents, with a threshold of 3 mm. Skewness was selected as the standard for grid quality evaluation. This standard is also known as grid distortion and its reference value varies from 0 to 1. The closer the value to 0, the better the grid quality. The meshing of this model corresponded to an unstructured hexahedral grid. The grid quality was controlled by the grid distortion standard. The maximum distortion range was 0.42–0.66, the weight was 0.24, the average value was 0.52 and the s.d. was 0.07. In order to improve grid quality and reduce computation cost, a grid independence analysis was carried out to determine the ideal choice of grid size. Figure 4 shows the model grids used are unstructured hexahedral. In order to improve grid quality and reduce computing cost, grid independence analysis is conducted to determine the reliability of grid size selection. The average deviation between sparse grid (i.e. 2.7 × 10^6^) and moderate grid (i.e. 4.7 × 10^6^) is about 6.15%. However, the average deviation between moderate grid and intensive grid (i.e. 6.7 × 10^6^) is only 0.90%. Therefore, the mesh generation method of moderate grid was applied in the present numerical simulation. Figure 5 displays the schematic diagram of the grid division. The number of grids varied between 4.68 × 10^6^–4.77 × 10^6^, with a mean of 4.72 × 10^6^ and s.d. of 3019. Considering the grid number and the maximum distortion, it can be concluded that the grid division met the simulation requirements.

### 2.3. Numerical methods and governing equations

According to modern fluid mechanics, the gas flow is described by three equations: the mass conservation equation, the energy conservation equation and the momentum conservation equation. The finite-element discretization method is applied to analyse the simulated fluid, and the analysed model body is discretized into small discrete units. The fluid equation is then solved by numerical simulation to generate physical parameters of the fluid.

The mass conservation equation can also be called the continuity equation, indicating the amount of volume and mass change caused by the density change of matter per unit volume per unit time,

2.1 $$\frac{\partial \rho }{\partial t}+\nabla \cdot (\rho v)=Sm.$$

The momentum conservation equation is the sum of all external forces acting on the computing unit over time; that is, the momentum change of the computing unit,

2.2 $$\frac{\partial }{\partial t}(\rho v)+\nabla (\rho vv)=-\nabla p+\nabla \cdot (\bar{\bar{\tau }}\mathrm{eff})+\rho g+F.$$

The energy conservation equation corresponds to the spatial accumulation of the resultant force on the computing unit; that is, the energy change of the computing unit. The velocity increase in the computing unit is the result of thermal pressure and surface force. Thus, the increment in velocity is equal to the heat flow gradient and surface force energy,

2.3 $$\frac{\partial }{\partial t}(\rho E)+\nabla \cdot (v(\rho E+P))=\nabla \cdot \left(k\mathrm{eff}\nabla T-\sum_{j}^{\;}hjJj+(\bar{\bar{\tau }}\mathrm{eff}\cdot v)\right)+Sh.$$

The corresponding temperature field in the greenhouse can be obtained using the discrete conservation equation and energy equation. Different researchers have used CFD to simulate the environment inside greenhouses in order to study various turbulence models. Nebbali *et al*. [21] and Santolini *et al*. [22] conducted comparative studies on different turbulence models, including Standard *k*-*ε*, RNG *k*-*ε* and Realizable *k*-*ε*. According to their results, the highest accuracy was obtained using the Standard *k*-*ε* turbulence model. Solar radiation plays an important role in greenhouse internal climate and internal airflow distribution, as follows:

2.4 $$\frac{\partial }{\partial t}(\rho k)+\frac{\partial }{\partial x_{i}}(\rho ku_{i})=\frac{\partial }{\partial x_{j}}\left[\left(\mu +\frac{\mu_{t}}{\delta_{k}}\right)\frac{\partial k}{\partial x_{j}}\right]+G_{k}+G_{b}-\rho \varepsilon -Y_{M}+S_{k}$$

and

2.5 $$\frac{\partial }{\partial t}(\rho \varepsilon )+\frac{\partial }{\partial x_{i}}(\rho \varepsilon u_{i})=\frac{\partial }{\partial x_{j}}\left[\left(\mu +\frac{\mu_{t}}{\delta_{\varepsilon }}\right)\frac{\partial \varepsilon }{\partial x_{j}}\right]+C_{1\varepsilon }\frac{\varepsilon }{k}(G_{k}+G_{3\varepsilon }G_{b})-C_{2\varepsilon }\rho \frac{\varepsilon^{2}}{k}+S_{\varepsilon },$$

where ρ corresponds to the fluid density (kg m^−3^); *k* indicates turbulent kinetic energy; *t* represents time (s); *µ_i_* and *µ_j_* are the instantaneous quantities (m s^−1^) in the direction of velocity *x_i_* and *x_j_*, respectively; *µ* is laminar dynamic viscosity; *µ_t_* indicates turbulent dynamic viscosity, which can be expressed as $\mu_{t}=\rho C_{\mu }(k^{2}/\varepsilon )$, where *C_µ_* is an empirical constant with the value of *C_µ_* = 0.85 and *ε* is the turbulent dissipation rate; *G_k_* corresponds to the turbulent kinetic energy *k* caused by the mean gradient of velocity; *G_b_* is turbulent kinetic energy *k* caused by buoyancy; Y_M_ indicates the effect of compressible turbulent pulsation expansion on the total dissipation rate; *S_k_* is a user-defined source item; *σ_k_* and *σ_ε_* are the reciprocals of the effective turbulent Prandtl number for turbulent kinetic energy *k* and dissipation rate *ε*; and *σ_k_*, *σ_ε_*, *C_1ε_* and *C_2ε_* are constants. According to the literature [23], *σ_k_* = 1.0, *σ_ε_* = 1.3, *C_1ε_* = 1.44 and *C_2ε_* = 1.92.

There are five major types of radiation models: Rosseland (R), P1, discrete transfer, surface to surface (S2S) and discrete ordinates (DO). With respect to the coupled method of energy balance, several experts have used the DO radiation model to describe the coupled convective and radiative exchange on the roof and walls of the greenhouse. The DO model may be directly applied to transparent, translucent and opaque optical media, such as air, thin films, glass and soil surfaces. It has been employed in climate models for greenhouses [24,25]. The heat transfer caused by the transparent film of the solar greenhouse was calculated using the DO model, which solved the radiative heat transfer in multiple discrete spatial directions.

During calculations, transient simulation was performed, and the analytical solution was obtained after 400 steps of calculation.

### 2.4. Boundary conditions and material properties

For calculation purposes, the south wind direction was taken as 0°, and the simulated wind speed was set as 1.5 m s^−1^. The airflow in the greenhouse displayed a large Reynolds number. This airflow involved a large area and also generated a vortex. Therefore, in the present study, the standard *k* − ε turbulence model was selected as the primary simulation method, and the solar radiation was imported by the solar ray tracing equation at the same time. In our model, the east, west and north sides of the greenhouse were set as the opaque wall boundary and the south roof was selected as the translucent boundary. Later, parametric modelling was performed. The physical parameters for soil, structure and air used in our model are shown in table 1. The detailed boundary conditions are described in table 2.

**Table 1.** Physical parameters used in CFD modelling of the greenhouse.

material	density *ρ* (kg m^−3^)	coefficient of thermal conductivity (W m^−1^ K^−1^)	specific heat capacity (J kg^−1^ K^−1^)
air	1.22	0.0242	1006.43
PE	923	0.38	2300
polystyrene	9	0.032	1246
soil	1900	2.0	2200

**Table 2.** Boundary conditions used in CFD modelling of the greenhouse.

boundary	property
inlet	velocity condition: 5 m s^−1^
outlet	pressure condition: 0 Pa
south roof surface	absorption coefficient: 0.15, reflectivity coefficient: 0.05, transmission coefficient: 0.80
north wall surface	absorption coefficient: 0.55, reflectivity coefficient: 0.45
north roof surface	absorption coefficient: 0.45, reflectivity coefficient: 0.55
side wall surface	absorption coefficient: 0.55, reflectivity coefficient: 0.45
soil surface	absorption coefficient: 0.40, reflectivity coefficient: 0.60
layer of frozen soil	constant temperature: 0°C

In the present study, ANSYS Fluent 18.0 was selected as the simultaneous calculation solver, and the three-dimensional transient analytical solution was obtained. The discrete format was performed using the second-order upwind style. SIMPLE solution was carried out, and Report was used to produce the data on cooling efficiency and wind speed. After calculation, each layer in the computational domain was rendered and the images were obtained.

## 3. Results

### 3.1. Validation of the numerical simulation

After boundary setting and preliminary calculation, the CFD simulation results were compared with the field measurements to verify the simulation accuracy of the established model. A temperature-humidity sensor was placed 10 cm below the soil as points 1, 2 and 3. The measurements were also performed in different planes (figure 2). Herein, measurements were taken at heights of 1 m, 3 m, 5 m and 5.5 m as points (a) 4–6, (b) 7–9, (c) 10–11 and (d) 12, respectively (figure 2). Our data indicated that simulation results were consistent with the actual measurements. Thus, the numerical simulation may be used to represent the real situation.

According to our data, the difference between the average simulated value and the average measured value at a height of 5 m was 1.4°C, with an average relative error of 3.5%. The largest average relative error was obtained at a height of 5.5 m. This position corresponded to the highest point of the greenhouse, which was also close to the top vent. Because of the gaps present in the greenhouse film, the temperature at position 12 was affected by external airflow. In our simulation, the external wind speed and direction represented the average values over a given period of time. This resulted in a large relative error between the average real-time measured values and the average simulated values. At a height of −0.1 m, the difference between the average simulated value and the average measured value was 0.94°C, with an average relative error of 3%. The surrounding areas of front and top vents were easily affected by the external airflow, leading to a large error during measurements. In this case, the average relative error was 8.3%.

Table 3 presents the average values obtained at the five heights selected for measurements in the greenhouse. As data indicated, the maximum difference between the measured values and the simulated values of the greenhouse was 3.5°C, the maximum relative error was 8.3%, the minimum difference was 0°C and the average relative error was 3.38%. Therefore, the changed pattern and the level of accuracy showed that our CFD model was effective. This means that the equations used in the model, the materials selected for the analysis of natural ventilation and the cooling control in the greenhouse based on different ventilation structures were all appropriate for the model. This model may be used for the analysis and simulation of the environment inside greenhouses subjected to different conditions. In summary, the simulation results of the solar greenhouse numerical model were consistent with the actual measurements. These data provide the theoretical basis for additional numerical simulations, which prove that our study is of practical application.

**Table 3.** Average measured and simulated values in the greenhouse.

measuring point height (m)	average measured value (°C)	average simulated value (°C)	average relative error value (%)
−0.1	30.06	31.59	5.08
1	41.00	41.00	0
3	48.46	49.89	2.95
5	56.25	58.12	3.32
5.5	55.49	58.50	5.42

### 3.2. Effect of environmental parameters on greenhouse ventilation

**Figure 6.**
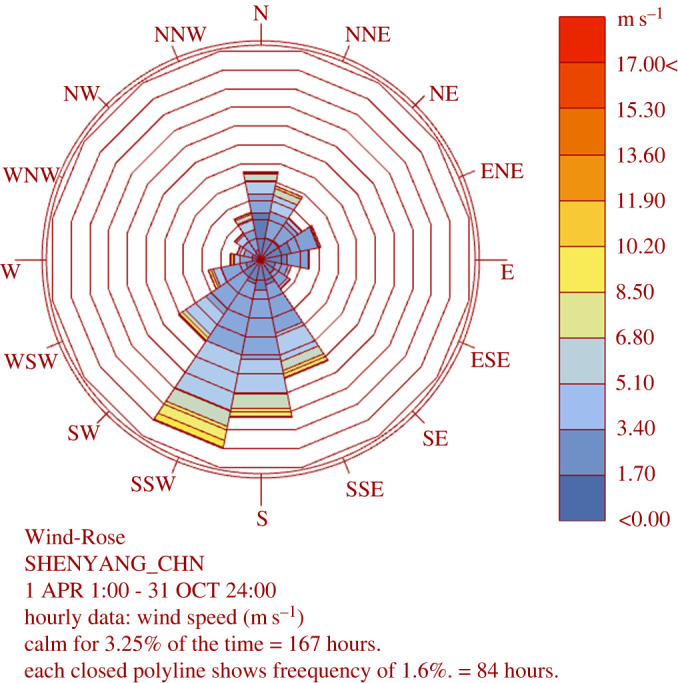
Wind rose of summer monsoon in Shenyang area.

Solar greenhouses require better ventilation during summer season. In the past, the greenhouse ventilation structure was a combination of bottom + top ventilation. However, this type of ventilation usually does not provide the conditions required for proper crop growth during summer. Rhino and Grasshopper were used to analyse the weather data of typical meteorological years in Shenyang to simulate the annual and summer wind speed in the area. The results are shown in figure 6. It demonstrated that in the summer in Shenyang, prevailing winds blew from the south, with an average wind speed of 1.5 m s^−1^. The simulation results were applied to the boundary conditions, where a fixed wind direction was selected for the external wind speed, which corresponded to 1.5 m s^−1^ in the SW direction.

### 3.3. Effect of different vent configurations on greenhouse ventilation

Natural ventilation does not provide the environmental conditions required for crop growth. Thus, in order to achieve a more efficient cooling system in the greenhouse, it is necessary to modify the ventilation structure. In the present research, the effect of newly added back roof ventilation on the cooling efficiency of the greenhouse was studied. In the corresponding design, a back roof vent displayed the same area as that of the top vent located in the middle of the rear roof of the greenhouse. Later, the cooling efficiency and the internal environment were simulated.

**Figure 7.**
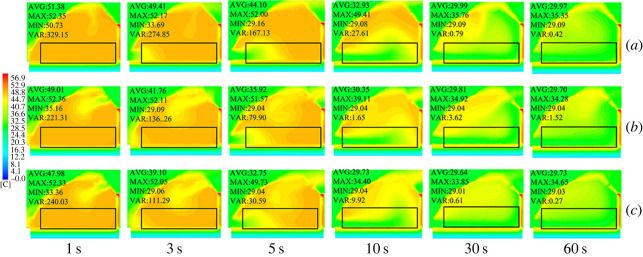
Contours of internal temperature field in greenhouses with (*a*) top, (*b*) back roof and (*c*) top + back roof ventilation systems.

In the present study, three different ventilation structures (structure A: FT; structure B: FB; structure C: FTB) were designed. These structures were used to simulate the cooling effect of the ventilation system on the greenhouse. The simulation results showed that when all vents were fully opened, the external cold air entered the greenhouse through the top vent as a result pressure. Since cold air has a high density and hot air has a low density, the cold air travelled to the ground in the greenhouse. This movement pattern was observed in the back roof ventilation structure in figure 7. As shown in this figure, the plant region has been highlighted in the contour and vector diagrams, which is 2 × 10 × 56 m^3^. Moreover, the statistical average value, maximum value, minimum value and the variance in the plant region are clarified. The wind pressure caused a large air vortex at the greenhouse ridge and back roof. This air vortex entered the greenhouse through the top vent because of the external cold air. Figure 7*a* shows the temperature distribution of a greenhouse with FT ventilation. As this figure shows, at a time of 3 s, the external cold air entered the front bottom of the greenhouse, causing a temperature reduction. In addition, in the FB and FTB ventilation systems, the external cold air entered the front bottom of the greenhouse after 5 s. Moreover, when the vent was opened, the external cold air entered the back roof, cooling the top air of the greenhouse (figures 7*b* and 6*c*). Taking into account the simulated temperature field inside the greenhouse, it can be concluded that the back roof ventilation system was effective in cooling the top part of the greenhouse, which was the point with the highest temperature.

**Figure 8.**
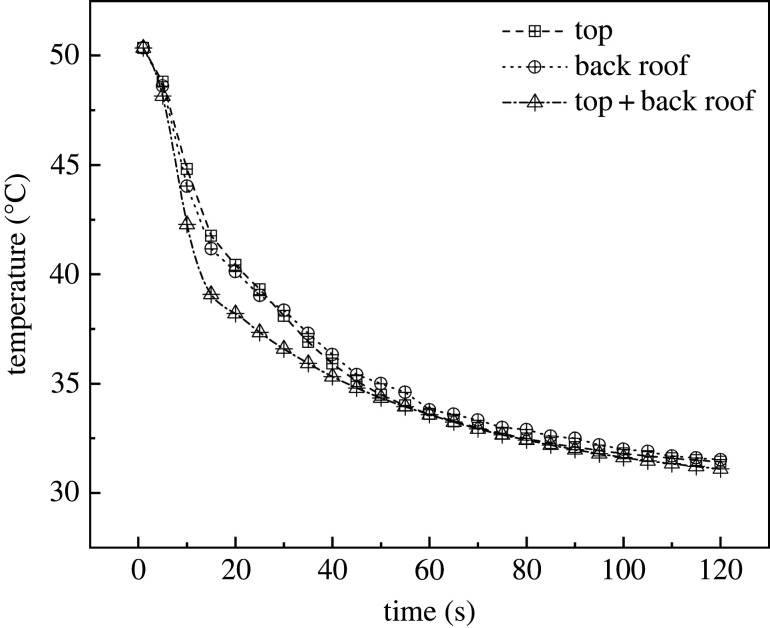
Internal cooling curves of greenhouses with (*a*) top, (*b*) back roof and (*c*) top + back roof ventilation systems.

The overall cooling efficiency of the greenhouse was analysed. The simulated data were obtained when the internal temperature of the greenhouse was equilibrated with the external temperature and vents were opened. The data showed that internal temperatures of the greenhouses with the three ventilation structures were stabilized at 100 s. The data of the first 120 s are displayed in figure 8. As shown, the cooling rates of FT and FB were similar. In addition, in FTB, the internal temperature was stabilized at 80 s after the vents were opened. This means that in this structure, the temperature stabilized 20 s before FT and FB. Also, in terms of cooling rate, FB showed a higher performance compared with FT during the first 5 s after the vents were opened. However, after 5 s, FT cooled faster than FB. Presumably, this occurred because during the first 5 s, external air directly entered the greenhouse from the back roof as a result of the air vortex, which allowed a faster exchange of the hot air present inside the greenhouse. According to these results, the best cooling structure for the greenhouse corresponded to FTB ventilation.

**Figure 9.**
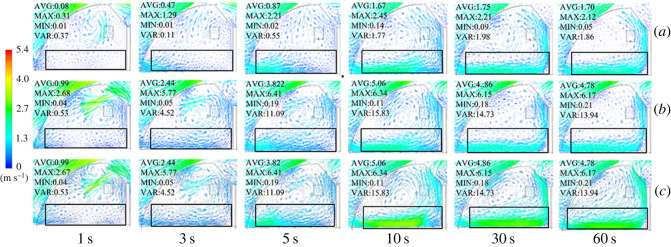
Contours of internal wind speed field of greenhouses with (*a*) top, (*b*) back roof and (*c*) top + back roof ventilation systems.

By simulating the internal wind velocity of the greenhouse with three different ventilation structures, the internal wind speed field was obtained (figure 9). As this figure shows, as the external air entered the greenhouse because of the air vortex outside the back roof, the wind speed reached 2.7 m s^−1^ in the first 3 s. In addition, in the three types of ventilation, the airflow inside the greenhouse reached a stable state after 60 s of ventilation. In the A structure, airflow entered the front bottom, travelled through the entire floor and moved upward at the back wall. When the airflow hit the top of the greenhouse, part of it was released, and another part travelled down the front roof and joined the ground airflow, forming a large vortex inside the greenhouse. This process corresponded to the mechanism of air exchange between inside and outside greenhouse when FT was used. The internal wind speed fields for FB and FTB were basically the same. At the moment the vents opened, the external air directly entered the back roof vents of the greenhouse. In addition, at a time of 5 s, the external air entered the greenhouse through the front bottom vents. Results also indicated that part of the airflow travelled over the entire greenhouse floor and moved upward at the back wall. Later, a portion of this airflow escaped from the back roof, and another portion flew upward until it was mixed with the air present at the top of the greenhouse. This caused the formation of a large air vortex inside the greenhouse, which contributed to the air exchange between the inside and outside of the greenhouse.

**Figure 10.**
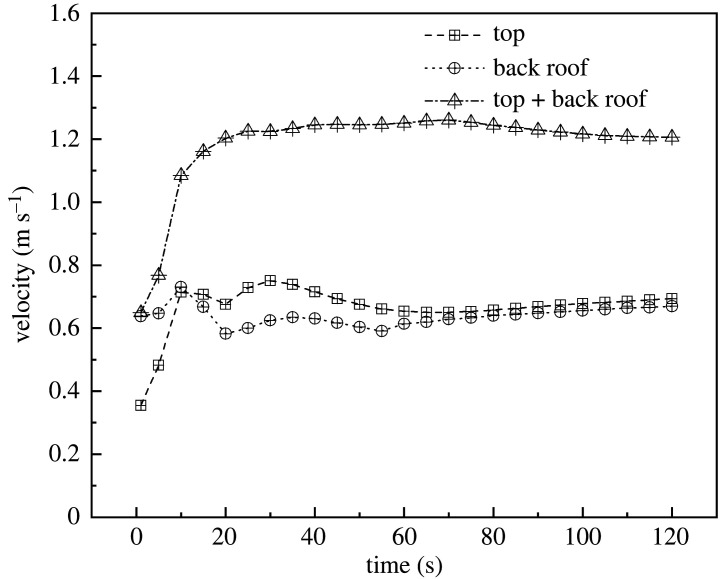
Internal wind speed using (*a*) top, (*b*) back roof and (*c*) top + back roof ventilation.

Furthermore, the overall wind speed inside the greenhouse was analysed. Data indicated that the three ventilation structures were stabilized at 60 s. Figure 10 displays the wind speed for the first 120 s. As this figure shows, when the internal wind speeds of structures A and B stabilized, the values were alike (approx. 0.6 m s^−1^). Consistent with the cooling rate, the internal wind speed in FTB was higher than those observed in FT and FB. In this case, wind speed stabilized at 30 s, which corresponds to 30 s quicker than FT and FB. A stable wind speed presented a value of 1.2 m s^−1^. The internal wind speed of FT was significantly higher than that in FB for the first 10 s. Afterwards, it was lower. A potential explanation for this phenomenon is that in FB, the air vortex in the back roof promoted the direct entrance of external air to the inside of the greenhouse. Therefore, according to our results, the optimal structure corresponded to FTB ventilation.

**Figure 11.**
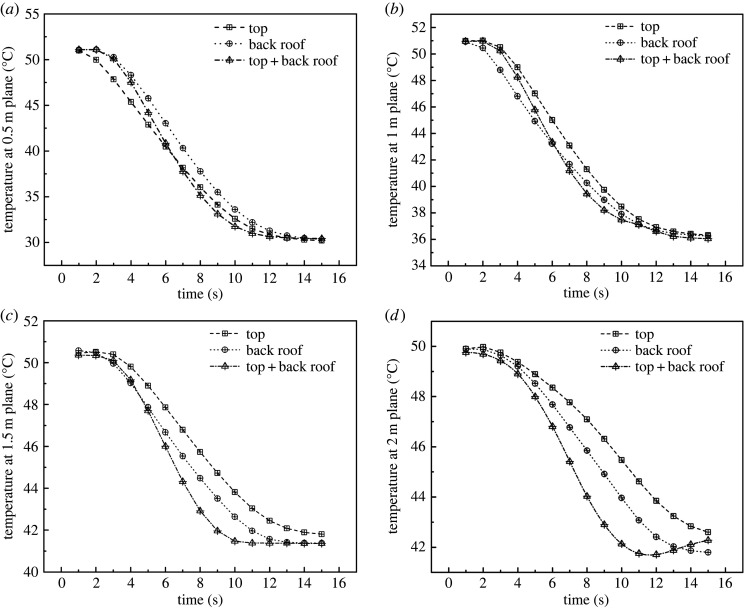
Temperatures at different heights inside the greenhouse with (*a*) top, (*b*) back roof and (*c*) top + back roof ventilation.

**Figure 12.**
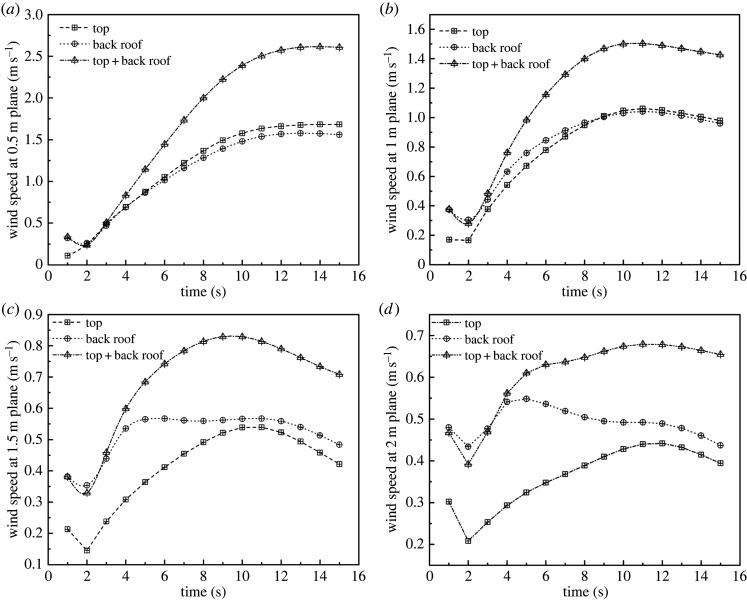
Internal wind speed curves at different heights with (*a*) top, (*b*) back roof and (*c*) top + back roof ventilation.

In general, the average canopy height is 2 m. For this reason, the optimal ventilation structure should take into account not only the overall cooling rate and wind speed, but also specific values at different height levels. Thus, considering the plant canopy, it is important to determine the cooling rate and wind speed at heights of 2 m and below. Therefore, the cooling rate and wind speed of the greenhouse were simulated at four horizontal planes (i.e. 0.5 m, 1 m, 1.5 m and 2 m). The simulated cooling curves are presented in figure 11 and simulated wind speed curves are in figure 12. As data indicated, in terms of cooling efficiency and internal wind speed, structure C displayed better performance compared with structures A and B in the four planes.

**Figure 13.**
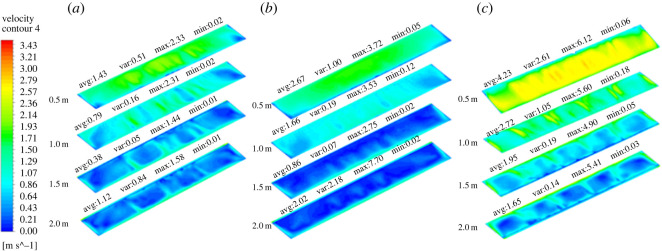
Contours of internal wind speed at different heights corresponding to different crop levels in greenhouse with (*a*) top, (*b*) back roof and (*c*) top + back roof ventilation.

In the present research, we also simulated the wind speed caused by different ventilation structures after the internal temperature stabilized at the above four horizontal planes. As shown in figure 13, FTB resulted in a more appropriate internal wind speed and plane uniformity compared with FT and FB. When the inside and outside temperatures reached equilibrium, FTB showed a faster wind speed on all planes compared with FT and FB. The maximum wind speed observed in FTB was 3 m s^−1^ at the 0.5 m plane, which may improve the ventilation of the plant canopy.

### 3.4. Effect of side vents on greenhouse ventilation

Some greenhouses include side vent structures. However, the ventilation effect of these side vents has not been systematically studied. In the present research, the cooling rate and the wind speed were simulated for greenhouses with and without side vents. The location of the side vents was selected at the ridge of the side walls, and their dimensions were 1.2 × 1.2 m^2^, since the fan had a size of 1.2 × 1.2 m^2^. Two symmetrical side vents were added to the side walls of the FTB roof ventilation structure.

**Figure 14.**
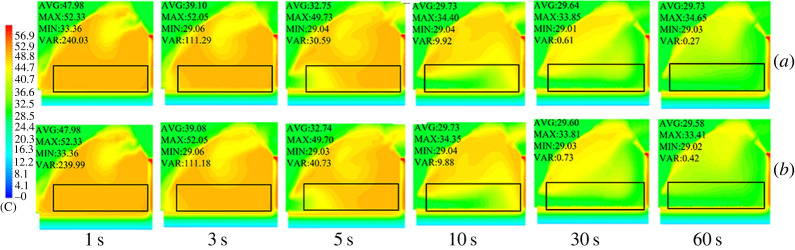
Contours of temperature inside the greenhouse (*a*) without side vents and (*b*) with side vents.

**Figure 15.**
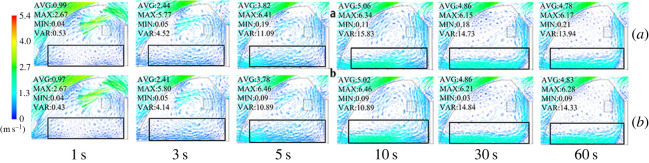
Contours of wind speed inside the greenhouse (*a*) without side vents and (*b*) with side vents.

The cooling of the greenhouse with (B) and without (A) side vents was simulated, and the temperature and velocity contours are shown in figures 14 and 15. According to our data, the side vents did not affect cooling or flow field inside the greenhouse during a ventilation time from 0 s to 120 s. After simulation of the thermal environment of a greenhouse with and without side vents, the air temperatures were plotted and analysed. Specifically, the average air temperature of the two different structures was plotted for different time sequences.

**Figure 16.**
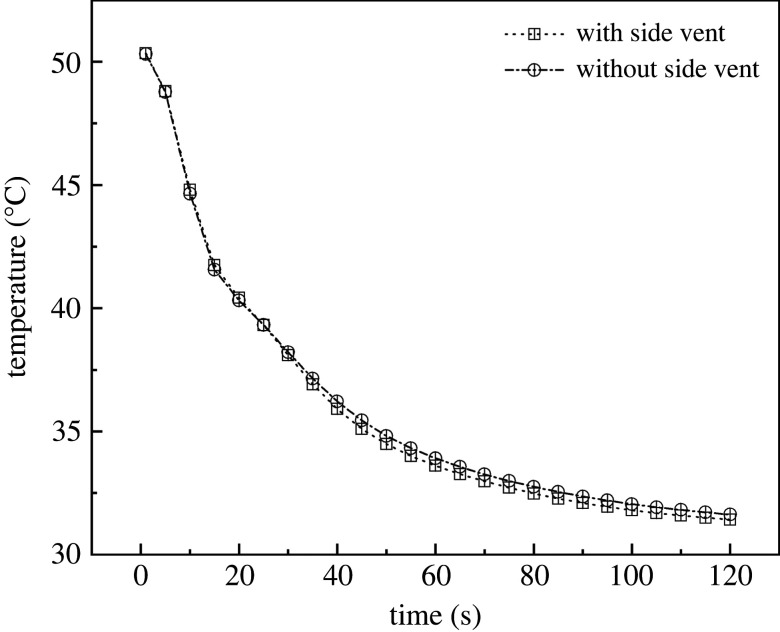
Internal cooling rate curves of greenhouses (*a*) without side vents (*b*) with side vents.

As figure 16 shows, between 1 s and 120 s, the cooling range and cooling rates resulting from the two configurations were approximately the same. The internal temperatures of the two types of greenhouses were both 51°C with vents closed. In addition, at the time of 30 s, the cooling rate of the greenhouse with side vents was slightly higher than that with no side vents. After this point, the greenhouse with side vents showed a slight advantage during the ventilation period. The temperature of the greenhouse with side vents reached equilibrium at 120 s and the value was only 0.45°C lower than that observed in the greenhouse with no side vents. Therefore, side vents had barely any effect on the cooling of the greenhouse.

**Figure 17.**
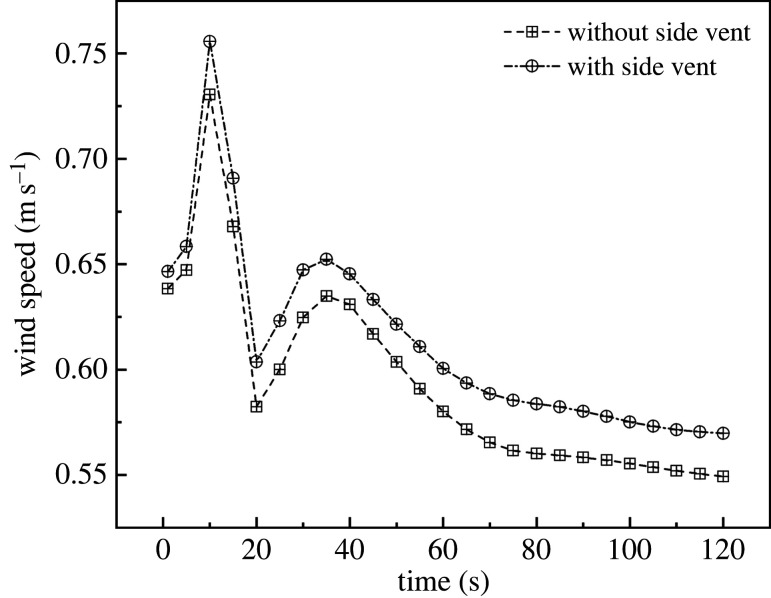
Internal wind speed curves for greenhouses (*a*) without side vents and (*b*) with side vents.

With respect to the simulation results of the overall wind speed with and without side vents, the air speed curves of these two treatments were plotted in time sequence. As shown in figure 17, the internal wind speed curves from 1 s to 120 s were quite different from the cooling curves for these two configurations. The internal wind speeds in the two greenhouses were 0.63–0.64 m s^−1^ at the time the vents opened and reached a maximum of 0.76 m s^−1^ within 10 s. After that, the greenhouse with side vents showed a small advantage. The wind speeds of both greenhouses stabilized at 100 s, with a value of 0.58 m s^−1^ for the one with side vents and 0.55 m s^−1^ in the case of no side vents.

**Figure 18.**
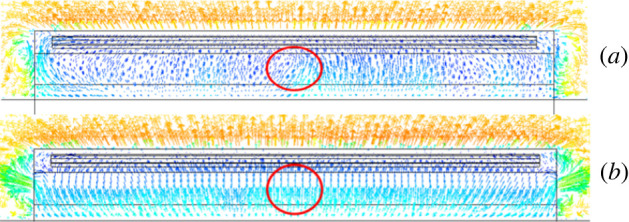
Contours of internal wind velocity field in greenhouses (*a*) without a side window and (*b*) with a side window.

A comparative analysis of these two greenhouses was carried out. As shown in figure 18, the velocity field for greenhouses with (B) and without (A) side vents demonstrated that these two configurations resulted in different ventilation effects. As marked by the red circle in figure 18, the air travelled uniformly in greenhouse B. For this reason, every section inside the greenhouse moved in an orderly manner toward the top vent. On the other hand, in the A configuration, the air moved towards the vent. However, this movement was not uniform, resulting in dissimilar airflow intensities in various sections of the greenhouse. In summary, a higher wind speed was observed in the middle of the greenhouse compared with other areas. According to these results, the ventilation system that includes side vents may be helpful in improving the ventilation uniformity inside the greenhouse. In conclusion, side vents increased the uniformity of greenhouse ventilation; however, they were not helpful in improving ventilation performance. For this reason, side vent ventilation is not recommended in a CSG.

To summarize, the ventilation performances of three vent structures are clarified in table 4. The cooling rate of FTB is significantly greater than that of FT and FB, showing a 24.84% and 5.52% improvement, respectively. The minimum temperature of FTB does not show obvious improvement over that of FT and FB. Nevertheless, the decrease of maximum temperature is significant. The average temperature of FTB was 13.81% and 3.65% lower than that of FT and FB. By contrast, a side window has little effect on the greenhouse ventilation; the improvement in cooling rate, wind speed and average temperature are only 0.52%, 2.09% and 0.11%, respectively.

**Table 4.** Quick comparison of ventilation performance in different configurations.

index	front bottom + top ventilation (FT)	front bottom + back roof ventilation (FB)	front bottom + top + back roof ventilation (FTB)	front bottom + top + back roof ventilation (FTB) with side vent
cooling rate	1.53	1.81	1.91	1.90
average wind speed	1.70	3.01	4.78	4.88
maximum temperature	46.17	44.06	42.83	42.61
minimum temperature	33.47	30.07	29.75	29.76
average temperature	39.63	36.09	34.82	34.78

## 4. Conclusion

In the present research, a novel FTB roof ventilation configuration for CSGs was proposed and different parameters under summer conditions were studied. Furthermore, the performance of the newly developed ventilation structure was compared with conventional structures that include front bottom and roof ventilation. The influence of the proposed ventilation structure on temperature and velocity distributions inside the greenhouse was investigated using field test and simulations. The results provide a theoretical basis for the design and optimization of the ventilation structure in solar greenhouses. The present study demonstrated that the optimal configuration corresponds to FTB ventilation without side vents. FTB stabilized the greenhouse temperature 20 s quicker than FT and FB. The comprehensive ventilation structure significantly reduced the formation of the air vortex inside the greenhouse and improved ventilation uniformity. In addition, this structure provided a higher ventilation flux, which improved the cooling rate, effectively increasing the ventilation of the plant canopy. The cooling rate of FTB showed a 24.84% and 5.52% improvement over FT and FB, respectively. Furthermore, the average temperature showed a 13.81% and 3.65% decrease, respectively. In addition, the present research proved that adding side vents to the CSG as an auxiliary ventilation strategy results in a more uniform ventilation inside the greenhouse, to a certain extent. However, it has little effect on the cooling rate. For this reason, the use of side vents for ventilation in a CSG is not recommended. The improvements in cooling rate, wind speed and average temperature are only 0.52%, 2.09% and 0.11%, respectively.

## Data Availability

The data are available from the Dryad Digital Repository: https://doi.org/10.5061/dryad.qnk98sfjr [26].
